# Male Carriers of the *FMR1* Premutation Show Altered Hippocampal-Prefrontal Function During Memory Encoding

**DOI:** 10.3389/fnhum.2012.00297

**Published:** 2012-10-30

**Authors:** John M. Wang, Kami Koldewyn, Ryu-ichiro Hashimoto, Andrea Schneider, Lien Le, Flora Tassone, Katherine Cheung, Paul Hagerman, David Hessl, Susan M. Rivera

**Affiliations:** ^1^Virginia Tech Carilion Research Institute, Virginia Polytechnic Institute and State UniversityRoanoke, VA, USA; ^2^Department of Psychology, Virginia Polytechnic Institute and State UniversityBlacksburg, VA, USA; ^3^McGovern Institute for Brain Research, Massachusetts Institute of TechnologyCambridge, MA, USA; ^4^Department of Psychiatry, Showa University School of MedicineSetagaya-ku, Tokyo, Japan; ^5^Medical Investigation of Neurodevelopmental Disorders Institute, University of California Davis Medical CenterSacramento, CA, USA; ^6^Veterans Affairs Medical Center, School of Medicine, University of California San FranciscoSan Francisco, CA, USA; ^7^Department of Biochemistry and Molecular Medicine, University of California DavisSacramento, CA, USA; ^8^Department of Psychiatry and Behavioral Sciences, School of Medicine, University of California DavisSacramento, CA, USA; ^9^Center for Mind and Brain, University of California DavisDavis, CA, USA; ^10^Department of Psychology, University of California DavisDavis, CA, USA

**Keywords:** fragile X premutation, memory, prefrontal cortex, psychophysiological interaction analysis

## Abstract

Previous functional MRI (fMRI) studies have shown that fragile X mental retardation 1 (*FMR1*) fragile X premutation allele carriers (FXPCs) exhibit decreased hippocampal activation during a recall task and lower inferior frontal activation during a working memory task compared to matched controls. The molecular characteristics of FXPCs includes 55–200 CGG trinucleotide expansions, increased *FMR1* mRNA levels, and decreased FMRP levels especially at higher repeat sizes. In the current study, we utilized MRI to examine differences in hippocampal volume and function during an encoding task in young male FXPCs. While no decreases in either hippocampal volume or hippocampal activity were observed during the encoding task in FXPCs, FMRP level (measured in blood) correlated with decreases in parahippocampal activation. In addition, activity in the right dorsolateral prefrontal cortex during correctly encoded trials correlated negatively with mRNA levels. These results, as well as the established biological effects associated with elevated mRNA levels and decreased FMRP levels on dendritic maturation and axonal growth, prompted us to explore functional connectivity between the hippocampus, prefrontal cortex, and parahippocampal gyrus using a psychophysiological interaction analysis. In FXPCs, the right hippocampus evinced significantly lower connectivity with right ventrolateral prefrontal cortex (VLPFC) and right parahippocampal gyrus. Furthermore, the weaker connectivity between the right hippocampus and VLPFC was associated with reduced FMRP in the FXPC group. These results suggest that while FXPCs show relatively typical brain response during encoding, faulty connectivity between frontal and hippocampal regions may have subsequent effects on recall and working memory.

## Introduction

The premutation expansion (55–200 CGG repeats) of the fragile X mental retardation 1 (*FMR1*) gene is linked to mood and other psychological symptoms (Franke et al., 1998; Johnston et al., 2001; Cornish et al., 2005; Hessl et al., 2005, 2007, 2011; Roberts et al., 2009), executive dysfunction (Cornish et al., 2005; Kogan and Cornish, 2010), primary ovarian insufficiency (POI; Allingham-Hawkins et al., 1999; Murray et al., 2000; Sullivan et al., 2005), and a late life neurodegenerative disorder characterized by intention tremor and gait ataxia, the fragile X-associated tremor ataxia syndrome (FXTAS; Hagerman et al., 2001; Jacquemont et al., 2003; Leehey, 2009; Garcia-Arocena and Hagerman, 2010). Neurocognitively, fragile X premutation carriers (FXPC) without FXTAS have been shown to exhibit deficits in several components of memory including declarative learning, working memory, and recall (Moore et al., 2004a; Cornish et al., 2005; Grigsby et al., 2008). Older FXPCs with FXTAS may also exhibit dementia (Bourgeois et al., 2007) and clinical reports from FXTAS patients or their caregivers suggest that memory deficiencies may precede or coincide with the motor degeneration (Bourgeois et al., 2006, 2007).

On a molecular level, the premutation allele results in increased *FMR1* mRNA levels and in some cases, decreases in fragile X mental retardation protein (FMRP; Tassone et al., 2000b; Kenneson et al., 2001). Increased *FMR1* mRNA is theoretically linked to neurodegeneration in a neurotoxic gain of function model for FXTAS and perhaps other clinical manifestations of the premutation (Hagerman and Hagerman, 2004a; Brouwer et al., 2009; Raske and Hagerman, 2009). FMRP also plays an important role in dendritic maturation (Chen et al., 2010) and the formation of axons and myelin (Greco et al., 2002, 2006). Thus, both increases in mRNA and decrements in FMRP levels may, both separately and in combination with each other, contribute to the premutation phenotype.

The hippocampus has one of the highest rates of *FMR1* transcription, especially in FXPCs, with higher levels of *FMR1* mRNA than many other areas of the brain (Tassone et al., 2004). Histological studies of post-mortem brain tissue from FXTAS patients have found high densities of intranuclear inclusions within the hippocampus (Greco et al., 2002, 2006). Further, Moore et al. (2004b) reported lower gray matter density in the amygdala-hippocampal complex in FXPCs compared to controls. Left hippocampal volume was also found to positively correlate with performance on delayed memory tasks in FXPCs (Jäkälä et al., 1997). However, subsequent structural MRI studies examining hippocampal volume have not found group differences between controls and FXPCs (Koldewyn et al., 2008; Adams et al., 2010).

Koldewyn et al. (2008) found that non-FXTAS FXPCs had lower hippocampal activation compared to controls during a recall task and this decrease was associated with increased *FMR1* mRNA levels. Moreover, male FXPCs, both with and without FXTAS, were found to demonstrate reduced frontal activity while engaged in a working memory task (Hashimoto et al., 2010), and *FMR1* mRNA levels negatively correlated with right inferior frontal cortex activity during the task in the combined FXPC group. Though it is difficult to specify the exact relationship between reduced frontal activity and memory ability in the task design used by Hashimoto et al. activity in prefrontal regions has been linked to memory encoding in many previous studies (Blumenfeld and Ranganath, 2007). The ventrolateral prefrontal cortex (VLPFC) contributes to successful memory formation, item specific encoding, and long-term memory formation (Wagner et al., 1998; Blumenfeld and Ranganath, 2006) while the dorsolateral prefrontal cortex (DLPFC) is active during successful encoding of items based on relational information (Murray and Ranganath, 2007; Blumenfeld et al., 2011).

The aim of the present study was to examine brain activation differences during an encoding memory task in a sample of young male FXPCs without FXTAS relative to controls. Based on the previous reports of memory and executive function problems in FXPCs, we hypothesized that, relative to controls, these individuals have lower hippocampus, parahippocampal gyrus, VLPFC, and DLPFC activation during memory encoding. In addition, activation of these regions was expected to correlate negatively with *FMR1* CGG repeat length and mRNA levels while positively correlating with FMRP levels.

## Material and Methods

### Participants

Participants included 24 men with the *FMR1* premutation (mean age 32.6 years) and 25 controls (mean age 30.1 years) matched on age, IQ, level of education, handedness, psychoactive medication use, and ethnicity. The Institutional Review Board at the University of California, Davis, approved the experimental protocol. All participants were informed of possible risks and consented in their enrollment of the study. *FMR1* DNA testing was used to confirm allele status for all participants. None of the participants included were mosaic for either repeat size or methylation. The CGG repeat sizes for FXPCs in the current study ranged from 54 to 199. Descriptive statistics for both groups are shown in Table 1. FXPC males were recruited through the screening of fragile X pedigrees of probands with fragile X syndrome. No participants were enrolled or referred to the clinic due to clinical symptoms. Controls were non-carrier males recruited or ascertained through postings in flyers, local newspapers, through a web-based psychology experiment sign-up site on the U.C. Davis campus, and through the MIND Institute Recruitment Core or were family members with a normal *FMR1* allele. Neurological assessments on all participants were normal, including absence of tremor and ataxia. Exclusion criteria included any acute medical condition such as renal, liver, or cardiac disease that may be associated with brain atrophy or dysfunction, migraine headache, seizure disorder, history of head trauma, toxic encephalopathy, encephalitis, or bacterial meningitis, history of alcoholism or drug problem, use of current medications that effect cerebral blood flow, and any conditions or implants that do not meet MRI safety guidelines.

**Table 1:** **Molecular genetic, demographic, and clinical descriptive statistics by group**.

	Control (*n* = 25)	Premutation (*n* = 24)	Significance
CGG repeats	28.32 (3.56)	104.71 (40.56)	*P* < 0.001
*FMR1* mRNA	1.46 (0.28)	3.57 (1.71)	*P* < 0.001
FMRP	100.38 (56.90)	77.08 (67.15)	ns
Handedness (RH)	80.0%	96.0%	ns
Psychoactive medication	33.3%	27.3%	ns
Age	30.12 (7.75)	32.58 (8.87)	ns
Caucasian	68.5%	83.3%	ns
Years of education	15.39 (3.60)	15.50 (2.89)	ns
Full scale IQ	125.52 (17.77)	117.92 (15.71)	ns


### Molecular genetic measures

#### CGG repeat size

Genomic DNA was isolated from peripheral blood lymphocytes using standard methods (Qiagen, Valencia, CA, USA). Repeat size and methylation status were determined with both PCR and Southern Blot analysis using an Alpha Innotech FluorChem 8800 Image Detection System (San Leandro, CA, USA) as detailed in (Tassone et al., 2008; Filipovic-Sadic et al., 2010).

#### *FMR1* mRNA

All quantifications of *FMR1* mRNA were performed using a 7900 Sequence detector (PE Biosystems) as previously described (Tassone et al., 2000a). Due to constraints in blood draw and storage, one FXPC was missing mRNA data.

#### *FMR1* FMRP

Fragile X mental retardation protein were quantified in lymphocytes from all subjects utilizing a recently described sandwich Enzyme Linked ImmunoSorbent Assay (ELISA) for FMRP (Iwahashi et al., 2009). The FMRP ELISA assay differs from the commonly used immunocytochemistry (IHC) method in that the ELISA approach provides a quantitative measure of FMRP level, whereas the IHC method does not measure protein level, only the proportion of cells with detectable staining. For quality control in the molecular data, outlier values of three interquartile range or greater from the median in were excluded from analysis to insure a normal distribution. Since the measure was added after the study had already begun, valid FMRP values were only available for a 39 participants (17 FXPC, 22 controls).

### MRI acquisition

A 3.0T Siemens scanner with Echo speed gradients and a Siemens 8-channel whole head coil was used to acquire images. The functional MRI (fMRI) sequence was performed using a single-shot gradient recalled echo planar imaging sequence that is auto corrected for motion and magnetic field distortions. It utilizes a point spread function mapping approach (Zeng and Constable, 2002) to correct images for distortions due to magnetic field inhomogeneities. The dimensions of the fMRI sequence include: 38 slices (3.4 mm thick), aligned 30° clockwise from parallel to the anterior and posterior commissure and covering the whole brain, a temporal resolution of 2 s using a *T*2* weighted gradient echo planar pulse sequence with TE 13 ms, flip angle 84°, FoV 220 mm, and base resolution 64. A high-resolution T1-weighted magnetization prepared rapid acquisition gradient echo (MPRAGE) sequence with TR 2170 ms, TE 4.82 ms, FoV 256 mm, 1.0 mm slice thickness, and 192 slices was acquired for the purpose of manual segmentation of the hippocampus and total cerebral volume (TCV) and to aid in localization, co-registration, and normalization of functional data. Structural and functional images were acquired in the same scan session.

### Hippocampal volume

Hippocampal volumes were quantified by operator-guided manual segmentation using Mayo BIR’s Analyze 8.5 and 9.0 (Robb and Barillot, 1989; Robb et al., 1989; Robb, 2001). These guidelines, used at the UC Davis MIND Institute Computational Neuroimaging Laboratory, were developed from the anatomical analysis of post-mortem human brains using histological sections of tissue cut perpendicular to the hippocampal axis. For a detailed description of this protocol, see Schumann et al. (2004). Hippocampal volumes were also corrected for TCV to account for overall individual differences in brain volume. The reliability intraclass statistic correlation between the two tracers on 10 cases was 0.934 for both the left and the right hippocampus.

### Total cerebral volume

To obtain a measure of TCV, each series of images was edited manually using Mayo BIR’s Analyze 8.5 and 9.0 (Robb and Barillot, 1989; Robb et al., 1989; Robb, 2001) to remove non-brain structures, the brainstem, and the cerebellum. Using a Gaussian cluster multispectral thresholding tool, the ventricles were defined and excluded. For a detailed description of this protocol, see Schumann et al. (2004).

### fMRI pre-processing

Pre-processing of the imaging data was completed using statistical parametric mapping software (SPM5; Wellcome Department of Imaging Neuroscience, University College London, UK). Images were corrected for movement using least squares minimization without higher-order corrections for spin history and normalized to stereotaxic Montreal Neurological Institute (MNI) space. Images were then resampled every 2 mm using fourth degree B-spline interpolation and smoothed with a 5 mm Gaussian kernel.

### fMRI encoding task

**Figure 1:**
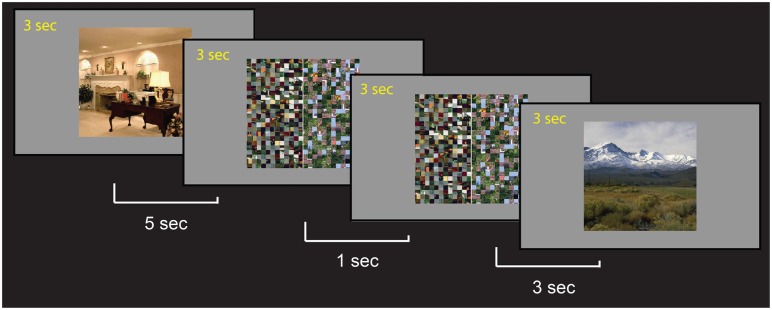
**Sample trials of the encoding task**. Participants were asked to memorize each scene and were told that they would be tested after the scan. During the scan, they indicated whether the scene was an indoor or outdoor picture. For the control scrambled picture trials, participants were asked to indicate if the patterns on the two sides matched.

In healthy controls, the hippocampus responds robustly during associative memory and encoding tasks (Killgore et al., 2000; Sperling et al., 2001, 2003; Stark and Squire, 2001; Yonelinas et al., 2001; Duzel et al., 2003; Giovanello et al., 2004). The encoding task was based on one used by Binder et al. (2005) designed to maximize bilateral hippocampal activation (Figure 1). Participants viewed two sets of stimuli. The first set consisted of complex color photographs of either indoor or outdoor scenes chosen from a commercial collection of digitized color photographs. Each picture in this set was presented only once. All words, persons, and animals were removed from pictures where necessary and images were cropped to 336 by 336 pixels. Participants were asked to indicate whether the picture was “indoor” or “outdoor” via two buttons with their right hand. The second set of stimuli consisted of scrambled versions of two of these photographs. The scrambled versions were created by segmenting the picture into 12-pixel square “tiles” and then scrambling the order of those tiles so that the pictures no longer contain discernible, meaningful shapes. These two pictures were then cut in half, and each half presented (with a white line between the two) with either its other half or with an exact duplicate. Participants were asked to identify whether the two halves exactly matched or not. Pictures from both sets (indoor/outdoor scenes and scrambled matching/non-matching pictures) were presented in a standard rapid-event-related design, each for 3 s. Picture order was pseudo-randomized and the intervals between pictures were jittered between 1, 3, and 5 s. One-hundred four pictures of each type were presented over two scanning runs, where runs lasted 10 min and 34 s. Participants were asked to pay close attention to the stimuli and told that they would be tested on their memory of the outdoor/indoor scenes subsequent to the scanning session.

Accuracy on the indoor/outdoor distinction and scrambled picture matching as well as reaction time data were collected. Immediately following scanning, participants completed a recall test where they were presented with all of the indoor/outdoor scene stimuli from the scan session plus 104 novel indoor/outdoor scenes. Participants indicated with a button press whether the picture was presented in the scanner or was novel. The combination of an event-related design and the post-scan test allowed us to examine brain function not only generally during encoding but also as a function of successful versus unsuccessful encoding. One FXPC participant’s post-scan recall was lost due to computer problems and was excluded from this performance analysis and subsequent functional contrast looking at correctly encoded trials versus incorrectly encoded trials.

The functional task was programmed using Presentation™ software on a Windows compatible computer. Initiation of scan and task was synchronized using a TTL pulse delivered to the scanner timing microprocessor board from a microprocessor connected to the computer. Stimuli were presented visually at the center of a screen at the participant’s feet using a custom-built magnet compatible projection system.

### fMRI analysis

Statistical analyses were performed on individual and group data using the general linear model and the theory of Gaussian random fields as implemented in SPM5 (Friston et al., 1995). For both within-group and between-group comparisons, significant clusters were defined as those that exceeded a threshold value equivalent to a one-tailed *t*-test at *p* < 0.01, Bonferroni corrected for multiple comparisons at the cluster level. For a higher level of quality control, a threshold value equivalent to a one-tailed *t*-test at *p* < 0.01 corrected for multiple comparisons using the False Discovery Rate (FDR) correction at the voxel level (Genovese et al., 2002) was used for all analyses. Once thresholded, activation foci were superposed on averaged normalized high-resolution MPRAGE images, their locations were identified both manually using an atlas with known neuroanatomical landmarks (Duvernoy and Bourgouin, 1999) and automatically using xjView (http://www.alivelearn.net/xjview8/).

A within-subjects procedure was used to model all the effects of interest for each subject by contrasting experimental and control trials (e.g., trials of scenes > trials of scrambled) and post-scan tests for successfully encoded trails (e.g., correctly encoded trials > incorrectly encoded trials). Models for individuals were identical across participants. This model estimates the error variance for each condition of interest across participants rather than across scans (Holmes and Friston, 1998). These contrast images were first generated on the individual level using a general linear model to determine voxel-wise *t*-statistics and creating one contrast image per participant, per effect of interest.

Within-group analyses of each contrast were performed to identify brain regions showing similar response modulations across participants in either the premutation or control groups for each contrast. Between-group analyses were then performed to determine differences in average activation responses to each contrast between the two groups.

Region of interest (ROI) analyses were carried out using MarsBar toolbox for SPM (Brett et al., 2002). Each contrast of interest was analyzed only in voxels that fell either within operator-guided manual segmentations of the individual specific hippocampus (applied to individual non-normalized functional images in native space) or MNI templates (Tzourio-Mazoyer et al., 2002) of areas of interest (applied to normalized images in MNI space). MNI templates used to investigate the current data included BA44, BA45, and BA47 within the VLPFC; BA9, and BA46 within the DLPFC; and parahippocampal gyrus. A *t*-statistic termed “contrast value” was then calculated as the average of all voxels that fell within the defined ROI. Contrast values reported for these analyses is comparable to the Z score reported in the whole-brain analysis tables. Contrast values were then used in between-group independent sample *t*-tests and correlation and regression analyses in conjunction with biological measures and neuropsychology data. For quality control in the neuroimaging data, outlier values of three interquartile range or greater from the median were excluded from analysis to insure a normal distribution. To correct for the 14 regions tested, Benjamini–Hochberg FDR was used to correct for multiple comparisons (Benjamini and Hochberg, 1995).

### Psychophysiological interaction analysis

An exploratory psychophysiological interaction (PPI) analysis (Friston et al., 1997) was performed to investigate neural correlations between the hippocampus and regions associated with memory encoding. The two seed regions used in the analysis were right and left hippocampi as defined by MNI templates. The first (physiological) regressor in the PPI analysis was the extracted time course from the seed regions de-convolved with a canonical hemodynamic response function (Gitelman et al., 2003). The second (psychological) regressor represented the contrast between the two condition types (1 for encoding trials and −1 for control scrambled trials). The PPI regressor was created by the interaction of the first and second regressor. A contrast image of the PPI regressor was generated from the fMRI time-series of each individual participant. Contrast values were extracted (for encoding versus control trials) that represented positive modulation of functional connectivity with the hippocampus and the following brain regions: the ipsilateral BA44, BA45, and BA47 of the VLPFC, BA9, and BA46 from VLPFC, and parahippocampal gyrus. The extracted mean values from each individual were used in group comparisons using SPSS. Benjamini–Hochberg FDR was used to correct for multiple comparisons (Benjamini and Hochberg, 1995) for the six regions.

## Results

Fragile X premutation carriers and controls did not differ significantly image, IQ, handedness, use of psychoactive medication, or level of education (see Table 1). As expected, the groups differed significantly on CGG repeat size [*t*(47) = −9.38, *p* < 0.001] and mRNA levels [*t*(46) = −6.06, *p* < 0.001]. FMRP was reduced by 23% in the FXPC group relative to controls (FXPCs: mean = 77.08, SD = 67.15; Controls: mean = 100.38, SD = 56.90); however this difference did not reach statistical significance, possibly as a result of high variability within both groups.

### Total cerebral and hippocampal volume

**Figure 2:**
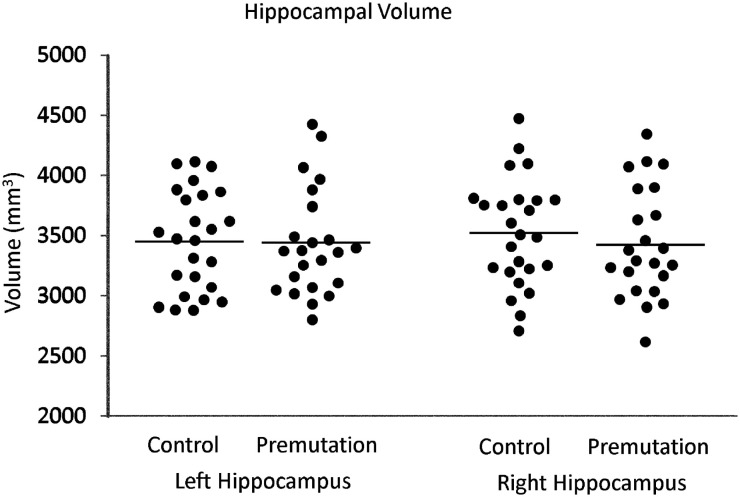
**Distributions of left and right hippocampal volume (corrected for total cerebral volume) in premutation carriers and controls**.

Hippocampal and TCV were extracted to examine possible neurodegeneration in FXPCs. Descriptive statistics for TCV and corrected hippocampal volumes are listed in Table 2. There were no significant differences between groups for TCV [*t*(47) = −0.522, *p* = 0.604] nor left [*t*(47) = 0.090, *p* = 0.929] or right [*t*(47) = 0.734, *p* = 0.426] corrected hippocampal volume (Figure 2). Raw volumetric measurements also did not differ between groups.

**Table 2:** **Descriptive statistics for total cerebral and hippocampus volumes by group**.

	Control(*n* = 25)	Premutation(*n* = 23)	Significance
Total cerebrum (mm^3^)	1,219,858 (17,096)	1,233,576 (20,042)	*P* = 0.604
Hippocampus* (mm^3^)			
Right	3528 (89)	3434 (92)	*P* = 0.467
Left	3461 (84)	3451 (89)	*P* = 0.929

*Corrected for total cerebral volume.

*Data shown are means ± 1 standard error*.

We hypothesized that hippocampal volume would negatively correlate with CGG and mRNA levels due to the neurotoxic gain of function model (Hagerman and Hagerman, 2004b; Brouwer et al., 2009; Raske and Hagerman, 2009). However, in the FXPC group, corrected hippocampal volumes were not significantly correlated with CGG repeat length, mRNA, or FMRP measures^1^.Thus, in the current sample of young to middle age adult FXPCs, as reported elsewhere (Koldewyn et al., 2008), we found no gross brain volume differences from typical controls and no volumetric differences within the hippocampus.

### Post-scanner recall results

In evaluating the performance on the encoding task, no significant difference between the groups was observed on post-scan recall task performance [FXPC mean percentage correct = 69.4%, control mean percentage correct = 69.8%, *t*(46) = −0.158, *p* = 0.875]. Both groups performed at close to 70%, showing that the encoding task was relatively difficult, and allowing us adequate numbers of incorrect trials for assessing brain activity differences on correct trials vs. incorrect trials.

### Hippocampus and associated brain activation

**Figure 3:**
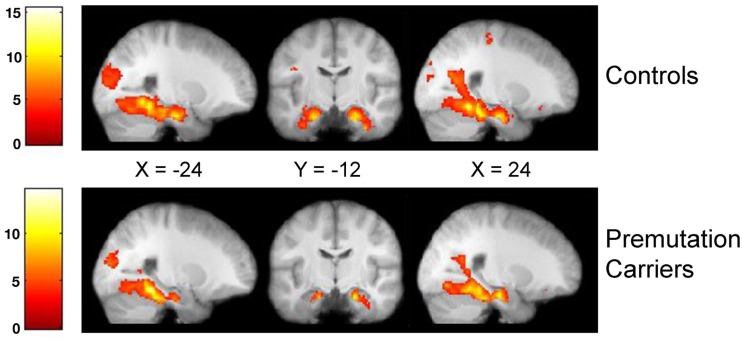
**Whole brain within-group analysis in the encoding > scramble trials for controls and premutation carriers**. Significant clusters were thresholded on the voxel level at *p* < 0.01 after FDR correction. Both groups significantly activated the bilateral hippocampus during this task. Bars at left represent degree of activation using the *t*-statistic.

To examine whether brain regions recruited during encoding for the FXPC group were less active than controls, we examined the BOLD signal between the FXPC and control group during all encoding trials (encode > scrambled) as well as during trials that participants later correctly recalled (correct > incorrect encoding). Qualitatively looking at within-group brain activity during the encoding task, the FXPC group activation patterns closely matched those of the control group (Table 3). Using a threshold of *p* < 0.01 with FDR correction for multiple comparisons at the voxel level, both groups showed robust activation of bilateral fusiform gyrus, parahippocampal gyrus, VLPFC, angular gyrus, lingual gyrus, and precuneus. The hippocampus was also significantly active bilaterally for both FXPC and control groups (Figure 3).

**Table 3:** **Within-group activation data for both premutation carriers and control group on the whole-brain level for the encoding > scrambled trials**.

Group	Area of activation	Cluster size	*Z* max	Peak coordinates
Controls	B angular gyrus	14078	7.52	26 −46 −12
	B cerebellum			
	B fusiform gyrus			
	B hippocampus			
	B lingual gyrus			
	B mid. and inf. occipital lobe			
	B parahippocampal gyrus			
	B precuneus			
	R mid. and B inf. temporal gyrus			
	Orbital frontal cortex	366	5.06	−8 42 −16
	L dorsolateral prefrontal cortex	282	4.29	−42 8 32
		54	3.8	54 −8 −22
	Posterior cingulum	265	5.68	0 −38 40
	L ventrolateral prefrontal cortex	102	5.36	−30 30 −22
	R precentral gyrus	86	4.21	24 −24 56
		30	0.001	64 −2 12
		42	0.001	56 0 24
	R dorsolateral prefrontal cortex	80	4.14	30 32 −14
	L postcentral gyrus	64	4.21	−54 −8 26
	L mid. and inf. temporal gyrus	43	3.97	−58 −8 −24
Premutation carriers	L cerebellum	4747	7.26	−32 −36 −22
	L fusiform gyrus			
	L hippocampus			
	L lingual gyrus			
	L mid. and inf. occipital lobe			
	L parahippocampal gyrus			
	L precuneus			
	R calcarine gyrus	4530	7.03	28 −46 −12
	R fusiform gyrus			
	R hippocampus			
	R lingual gyrus			
	R mid. and inf. occipital lobe			
	R mid. and inf. temporal gyrus			
	R parahippocampal gyrus			
	R precuneus			
	R ventrolateral prefrontal cortex	65	4.75	32 34 −16
	L ventrolateral prefrontal cortex	55	5.04	−32 32 −18

*The thresholding is set at *p* < 0.01 with FDR correction and number of voxels in a cluster >25*.

The between-group comparison across the whole brain for the encode > scrambled contrast revealed no significant activation differences in either direction at *p* < 0.01 with Bonferroni correction at the cluster level. A ROI analysis of average voxel signal extracted from participant-specific manually segmented hippocampi again did not reveal significant differences between the groups [right hippocampus: *t*(46) = 1.454, *p* = 0.153; left hippocampus: *t*(45) = 1.372, *p* = 0.421].

The results of the post-scan recall test were used to create a model allowing us to examine brain activity during trials in which the stimulus was correctly encoded (i.e., subsequently remembered) compared to trials in which the stimulus was not encoded (subsequently not remembered). Using a threshold of *p* < 0.01 voxel threshold and a Bonferroni correction at the cluster level, both groups showed similar areas of activation including areas such as DLPFC, parietal regions, middle temporal gyrus, and fusiform gyrus (Table 4).

**Table 4:** **Within-group activation data for both premutation carriers and control group on the whole-brain level for the correctly recalled > incorrectly recalled trials**.

Group	Area of activation	Cluster size	*Z* max	Peak coordinates
				
Controls	R angular gyrus	2365	4.69	28 −62 26
	R mid. and sup. occipital lobe			
	R mid. temporal gyrus			
	R precuneus			
	L mid. and sup. occipital lobe	806	4.5	−38 −82 24
	R fusiform gyrus	554	4.35	24 −42 −18
	R dorsolateral prefrontal cortex	464	4.29	46 30 12
	R inf. temporal gyrus	253	3.98	44 −64 −10
	L dorsolateral prefrontal cortex	559	3.93	−50 14 26
	L fusiform gyrus	404	3.67	−28 −42 −14
	Orbital frontal cortex	188	3.56	8 46 −14
Premutation carriers	R angular gyrus	6144	5.2	36 −76 30
	R fusiform gyrus			
	R hippocampus			
	R inf. parietal gyrus			
	R lingual gyrus			
	R mid. and inf. temporal gyrus			
	R occipital lobe			
	R parahippocampal gyrus			
	L fusiform gyrus	6636	5.07	−30 −38 −18
	L hippocampus			
	L lingual gyrus			
	L mid. and inf. temporal lobe			
	L occipital lobe			
	L parahippocampus			
	L precuneus			
	L dorsolateral prefrontal cortex	727	4.6	−40 6 22
	R ventrolateral prefrontal cortex	382	4.29	30 34 −12
	R dorsolateral prefrontal cortex	313	4.01	48 32 16

*The thresholding is set at *p* < 0.01 with Bonferroni correction on the cluster level*.

In the between-group comparison for the correct > incorrect encoding contrast, however, there were no significant activation differences on the whole-brain level with *p* < 0.01, with Bonferroni correction at the cluster level. Using a hippocampal ROI analysis, there were also no significant differences between FXPCs and controls [right hippocampus: *t*(45) = −0.065, *p* = 0.948; left hippocampus: *t*(44) = −0.608, *p* = 0.547]. The FXPCs did show greater signal in left BA44 of the VLPFC than the controls [*t*(46) = −2.033, *p* = 0.048], but this result did not remain significant after correction for multiple comparisons.

### Molecular and functional MRI correlation analysis

In the FXPC group, CGG repeat number was strongly and positively correlated with *FMR1* mRNA expression (*r* = 0.918, *p* < 0.001). There was a negative correlation between mRNA and FMRP measures that approached significance (*r* = −0.427, *p* = −0.099).To test the effects of these molecular measures on brain activations, a correlation analysis was run between the BOLD signal from regions associated with encoding and all molecular measures.

Hippocampal activation did not significantly correlate with any molecular measure in the encoding > scramble control contrast in the FXPCs. After corrections for multiple comparisons, FMRP levels were negatively correlated with activation in the left parahippocampal gyrus (*r* = −0.672, *p* = 0.003). At the uncorrected level, mRNA level was negatively associated with right BA44 (VLPFC; *r* = −0.452, *p* = 0.035) and FMRP was negatively correlated with right parahippocampal gyrus (*r* = −0.565, *p* = 0.018) and right BA46 (DLPFC; *r* = −0.591, *p* = 0.012) activation. Post-scan behavioral recall results did not show any significant correlations with ROI results in each group. The control group did not have enough variance in the CGG repeat numbers and mRNA levels for the analysis. There were also no significant brain activations that correlated with FMRP that survived the correction for multiple comparisons in the control group.

For the correct > incorrect encoding contrast, mRNA level was negatively correlated with activation of the left BA9 of the DLPFC (*r* = −0.656, *p* < 0.001) in the FXPC group after correction for multiple comparisons. The control group lacked the variance in mRNA levels necessary to test for the same correlation. Several other regions showed some correlation within the FXPC group, but did not pass the stringent correction for multiple comparisons, including: CGG repeat size with right BA44 of the VLPFC (*r* = −0.504, *p* = 0.017) and with left BA9 of the DLPFC (*r* = −0.483, *p* = 0.020). In addition, mRNA level was associated with reduced right BA44 activation (*r* = −0.517, *p* = 0.016) and right BA9 activation (*r* = −0.552, *p* = 0.008) at the uncorrected level. Lastly, FMRP correlated positively with left BA44 activation (*r* = 0.512, *p* = 0.043) at the uncorrected level.

### Functional connectivity analysis

**Figure 4:**
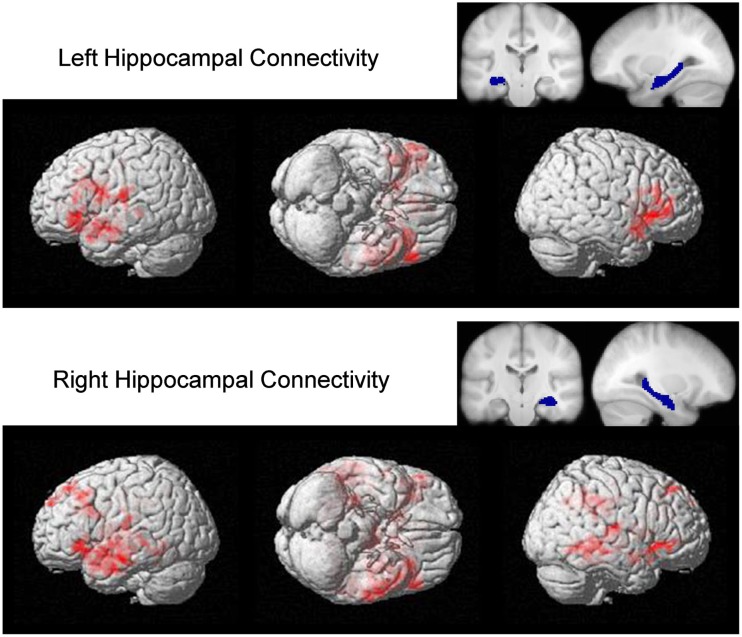
**Whole brain between group PPI analysis showing activation connectivity greater for controls than premutation carriers**. The analysis was seeded in the left and right hippocampus (the seed regions is displayed with corresponding blue masks). Significant clusters, displayed with red clusters, are thresholded on the voxel level at *p* < 0.05 with cluster size >500.

The molecular effects that are associated with the fragile X premutation have been shown to influence and change brain connectivity through changes to synaptic connections (Bear et al., 2004; Bassell and Warren, 2008). To test if functional connectivity within regions associated with encoding is affected in FXPCs, we used a PPI analysis for both between-group comparisons and within-group correlations. Both hippocampi (MNI template) were used as seed ROIs to test for encoding dependent connectivity. When seeded for the right hippocampus, the FXPC group had significantly lower connectivity with right BA47 of the VLPFC [*t*(46) = 2.490, *p* = 0.016] and right parahippocampal gyrus [*t*(47) = 3.777, *p* < 0.001] than the control group after correcting for multiple comparisons. At the uncorrected level, several significant group differences were observed, including lower right hippocampal co-activations with right BA46 of the DLPFC [*t*(47) = 2.270, *p* = 0.028] and lower left hippocampal co-activations with left BA47 of the VLPFC in the FXPC group than in the controls (See Figure 4; Table 5).

**Table 5:** **Between-group comparisons of encoding task dependent functional coupling (PPI) with the hippocampi as seeded regions**.

Seeded region	Coupled region	Controls	Premutation carriers	Significance
Left hippocampus	L BA 47 (VLPFC)	0.0120	−0.2423	*p* = 0.037
Right hippocampus	R BA 47 (VLPFC)*	0.0419	−0.2353	*p* = 0.016
	R BA 46 (DLPFC)	0.1571	−0.1100	*p* = 0.028
	R parahippocampal gyrus*	0.2438	−0.0950	*p* < 0.001

*Average intensity levels shown were extracted for each participant using individually non-normalized, individually traced hippocampal masks using Marsbars toolbox (Brett et al., 2002)*.

*Denotes significant between-group co-activation differences when corrected for multiple comparisons

**Figure 5:**
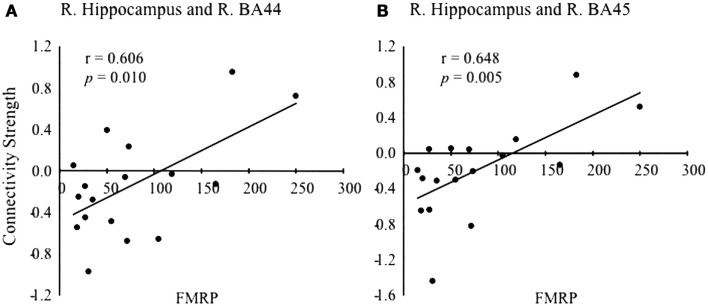
**Scatter plot showing the correlation between fragile X mental retardation protein (FMRP) levels and the strength of connectivity between the right hippocampus with right BA44 (A) and right BA45 (B) of the VLPFC**.

Connectivity between right hippocampus and right BA44 (*r* = 0.606, *p* = 0.010) and right BA45 (*r* = 0.648, *p* = 0.005) was positively correlated with FMRP levels after adjusting for FDR correction for multiple comparisons (Figure 5) for the FXPCs, only. At the uncorrected level, mRNA levels were positively correlated with the connectivity between the right hippocampus and right parahippocampal gyrus (*r* = 0.514, *p* = 0.012) and FMRP levels were positively correlated with right hippocampal connectivity with and BA47 (*r* = 0.524, *p* = 0.037). The functional connectivity results did not correlate with post-scan behavioral recall results.

## Discussion

The first aim of the current study was to investigate hippocampal differences during the encoding of complex scenes in young and middle aged male FXPCs with no symptoms of FXTAS. In contrast to our previous findings in this population during a recall task (Koldewyn et al., 2008), we did not observe any reductions in hippocampal activity during encoding. It is important to note, however, that these findings are not contradictory as memory encoding and memory recall are separate processes (Prince et al., 2005; Kim et al., 2010). The current results are also consistent with previously reported results showing typical hippocampal activity during the encoding phase of a continuous working memory task (Hashimoto et al., 2010) in male FXPCs. By using an encoding task in the present study, we aimed not only to examine whether the hippocampal hypoactivation we observed during recall would also be seen during encoding but also if the hippocampus would show hypoactivation in a task specifically designed to maximize its activity. While the main contrast of interest (all encoding trials > control trials) might therefore show “ceiling” effects in the hippocampus for both groups, we expected that a contrast looking at correctly encoded trials > incorrectly encoded trials would have the sensitivity to show any between-group differences in hippocampal response during encoding. No such between-group differences were observed for either contrast, nor did we see differences in memory performance on a post-scan recall test. Despite this, the current results suggest that elevated *FMR1* mRNA levels and decreased FMRP levels seen in FXPCs *do* affect encoding. *FMR1* mRNA levels appear to play a role in reduced activity in frontal regions during correctly encoded while FMRP levels are related to decreased parahippocampal gyrus activity during all encoding trials.

Encoding, especially of complex scenes, has been associated with brain regions other than the hippocampus. A second aim of the current study was to examine molecular correlates in regions associated with encoding beyond the hippocampus, including parahippocampal cortex (Powell et al., 2004), DLPFC (Murray and Ranganath, 2007; Blumenfeld et al., 2011), and VLPFC (Wagner et al., 1998; Blumenfeld and Ranganath, 2006). Relevant to the particular encoding task used in the current study, the parahippocampal gyrus, especially a region called the parahippocampal place area, is thought to be specifically engaged in scene processing and plays a role in encoding scenes into memory (Epstein et al., 1999). Anatomically, the hippocampus is also directly connected to the parahippocampal gyrus (Powell et al., 2004). Interestingly, right parahippocampal activation correlated negatively with FMRP levels in the FXPC group only. FMRP is a regulatory protein for several pathways, including group 1 metabotropic glutamate receptors (mGluR) that are involved in long-term depression (LTD) of transmission at hippocampal synapses leading to specific memory formation. Increased mGluR function from decreased levels of FMRP is thought to produce elongated and immature dendritic spines in the hippocampus (Bear et al., 2004), which have been found in autopsies of full mutation fragile X carriers (Irwin et al., 2001). Reduced FMRP has also been shown to abnormally increase LTD, a process critical for learning and memory storage, in the mouse hippocampus (Bear et al., 2004). It is important to note that while FXPCs do not have the same deficits in FMRP production as those with the fragile X full mutation, partial reduction in FMRP may still result in disruptions in mGluR production. The premutation mouse model displays lower hippocampal neuron viability compared to wild type mice (Chen et al., 2010) with changes seen not only in neuronal conformation and dendritic maturation, but also in synapse formation, axonal guidance, and neural circuits (Bassell and Warren, 2008). One speculation is that as FMRP levels decrease in FXPCs, task-related activation in the parahippocampus increases in an effort to compensate for the lack of synaptic maturity.

Consistent with previous research in healthy controls (Paller and Wagner, 2002), the VLPFC was more active during encoding for items that were later remembered than items that were forgotten in both FXPC and control groups. The DLPFC was also significantly more active during correctly encoded trials than during incorrectly encoded trials for both groups. Activity in DLPFC has been associated with successful memory formation in typical subjects (Staresina and Davachi, 2006; Murray and Ranganath, 2007), especially in encoding relational information between items (Blumenfeld and Ranganath, 2006). There were no group differences in activation between FXPCs and controls in either DLPFC or VLPFC for correctly remembered trials. However, in the FXPC group, left DLPFC activity was negatively correlated with *FMR1* mRNA levels during correctly remembered trials. At the uncorrected level, right DLPFC and right VLPFC both showed negative correlations with mRNA level, and FMRP level positively correlated with left VLPFC activation; results that were in line with our hypothesis of possible neurodegeneration with increased mRNA based on the neurotoxic gain of function model (Hagerman et al., 2004; Brouwer et al., 2009; Raske and Hagerman, 2009) despite the fact that they did not survive the FDR correction for multiple comparisons. FXPCs with abnormal molecular measures compared to the controls (i.e., low FMRP and high mRNA) did not show the increase in frontal activation.

Our fMRI results do not support the idea that the hippocampus is generally dysfunctional in young adult male FXPCs. Instead, the results of an exploratory PPI connectivity analysis revealed that FXPCs exhibited significantly less co-activation between right VLPFC and the right hippocampus than the control group. The strength of the co-activation between the right hippocampus and right VLPFC also positively correlated with FMRP levels in the FXPCs, but not in the controls. The VLPFC is very important for successful memory encoding (Hampshire et al., 2007), controlled selection of goal-relevant item information, and specific and relation encoding (Blumenfeld et al., 2011). The finding of reduced connectivity between the VLFPC and the hippocampus, especially in light of the correlation between the strength of this connectivity and FMRP levels, suggests that reduced encoding efficiency in the FXPCs may result from differences in hippocampal connectivity. Reductions in functional connectivity in FXPCs was also evident on a local level as correlated activity between the parahippocampal gyrus and the hippocampus was significantly reduced in FXPCs compared to controls. Given that there are direct structural connections between parahippocampal gyrus and the hippocampus that play an important role in memory formation (Powell et al., 2004; Van Strien et al., 2009), it stands to reason that the observed changes in functional connectivity may also contribute to memory differences in the FXPCs. These differences may only be observable when measuring hippocampal activation during recall (Koldewyn et al., 2008).

Human neuroanatomical studies of male FXPCs with FXTAS have found neurodegeneration in frontal white-matter (Brunberg et al., 2002; Greco et al., 2002, 2006). Even though participants in this current study were adult FXPCs without symptoms of FXTAS, it can be expected that some may eventually develop FXTAS and could currently have prodromal stages of neurodegeneration, as suggested by Loesch et al. (2008) Longitudinal studies are needed to examine these questions. A direct examination of either *structural* connectivity or overall white-matter integrity was not part of the current study, but could be a fruitful direction for future research.

There were several important limitations to this study. Although we attempted to minimize participant recruitment bias through random selection of premutation carriers from pedigrees, it remains possible that more (or less) affected individuals than represented by the whole population of premutation carriers may choose to participate in the study. About a third of the participants in the FXPC group were on psychoactive medication, which may provide alternative explanations to our results and may lower the generalizability in our findings. Genetic measures used in the analysis were extracted peripherally and may not represent the concentration found in brain tissues. Valid FMRP values were only available for a subsample of our participants since the measure was added after the study had already begun; thus correlations with FMRP must be considered preliminary. Lastly, results from the exploratory PPI connectivity analysis should be considered preliminary as well, as the task was not designed specifically for connectivity analysis and maybe underpowered due to the small sample size.

The goal of the current study was to examine brain activity in the FXPCs during an encoding task. In addressing our primary hypotheses, we did not find reduced hippocampal or frontal activation in a between-group analysis, nor did the FXPC group show performance or structural differences compared to controls. On the other hand, we did find molecular correlations with both frontal and parahippocampal activations in the FXPC group. These findings, along with research on the roles of FMRP and mRNA, suggest that differences in hippocampal connectivity during encoding may lead to subsequent deficits in recall reported in a previous study (Koldewyn et al., 2008). Our hypothesis was supported with an exploratory PPI connectivity analysis showing lower functional connectivity between the right parahippocampal gyrus and right VLPFC in FXPCs than controls. Furthermore, this reduction in connectivity was positively correlated with decreases in FMRP level in the FXPC group. Our results suggest that while FXPCs show a relatively typical hippocampal response during encoding, faulty connectivity between frontal and hippocampal regions observed may have subsequent effects during recall. Perhaps a combination of mRNA and FMRP affects both brain activity and connectivity in systems important for the encoding of memory. Such differences might be exacerbated in an older population, especially those that later develop FXTAS.

## Conflict of Interest Statement

The authors declare that the research was conducted in the absence of any commercial or financial relationships that could be construed as a potential conflict of interest.

## Footnotes

^1^CGG repeat length and mRNA measures do not have high enough variance within the control group for meaningful correlation analyses and FMRP measures did not correlate with hippocampal volumes in the control group.
